# Notes on Plague in Bristol in 1916

**Published:** 1917-04

**Authors:** D. S. Davies

**Affiliations:** Medical Officer of Health, Bristol


### NOTES ON PLAGUE IN BRISTOL IN 1916.

BY

LIEUT.-COL. D. S. DAVIES, R.A.M.C. (T.),

*Medical Officer of Health, Bristol.*

SINCE the recrudescence of plague in 1894 the Port of Bristol was threatened in 1901, 1902, and 1909, but upon each occasion the infection amongst rats was not permitted to reach shore, and as ship-borne rat infection is the usual channel of introduction, watch over arrivals in the Port has been unceasing and vigilant.

Early in August, 1916, the disease unexpectedly appeared in the centre of the city, independently of Port infection, in a rag warehouse receiving no imported rags, but collecting only from adjacent counties.

Four cases in all occurred, all of which were directly connected with this warehouse, which contained two hundred tons of rags, infested by rats and swarming with fleas. All the cases untimately recovered. Sixty-one rats were caught, alive or dead, on the premises, and of these six (three caught alive, three found dead) were definitely proved to have plague.

As the warehouse and its contents were obviously the focus of infection, it was decided to destroy by fire the whole of the contents. This necessitated elaborate precautions. The issue of rags was at once prohibited and work stopped. Food was supplied to keep the rats from migrating, while a staff of twenty-nine were inoculated against plague. When the staff commenced work, the bales of rags were first carefully netted round to prevent escape of rats, were then drenched with disinfectants, and conveyed in closed vans to one of the city destructors ; each bale had to be weighed and allowance made for weight of disinfectant, to estimate compensation. The work took ten days, sixteen tons of disinfectant were used, and two tons of lime.

Each person employed was bathed and his clothes baked after each day's work before returning home, to avoid carriage of infection by fleas ; the Health Committee's workmen were temporarily housed at the Central Disinfecting Station during this period. All persons employed at the warehouse were kept under supervision for six weeks, and affected houses and inmates were thoroughly dealt with to destroy all fleas, as bubonic plague in exclusively spread by these parasites.

A large area surrounding the district was mapped out, inspected in detail, and rat-catching systematically carried out for six months ; special attention was paid to rat-proofing the basements and to removal of wall food ; the riverside districts and the Port of Avonmouth were also searched for rats ; over 9,000 rats were caught, and of these nearly two thousand were examined at the University laboratory for plague. No plague rats were found in the Port or City other than those actually in the warehouse itself, and no further cases have occurred. No explanation has been forthcoming of this one-spot-infection in the centre of the city, which may have been accidental or intentional.

One interesting point is that on one of the plague rats in the warehouse, an ordinary brown or Norwegian rat, in place of this rat's proper flea (*Ceratophyllus fasciatus*) there was found the flea (*Xenopsylla Cheopis* (Rothschild)) which properly belongs to the Indian black rat (*Mus Rattus*), and which is known as the plague flea. The Hon. Charles Rothschild, who confirmed this fact, points out that this species is a scarce vagrant to the British Isles, usually introduced by Port rats, and considers that the occurrence of this tropical flea in the British Isles is of great interest to entomologists and pathologists.

How did this tropical flea gain access to an English rat in the middle of an English city? Russell states that direct experiment has proved that a flea may remain infective for fifteen days, while Raybaud states that a flea may hibernate for a month without nourishment, during which time plague germs may persist unharmed in its stomach. Rosenau in quoting this statement adds: "This fact may be of importance for the transmission of plague to a distance."

